# Urgent Airway Management and Postoperative Complications in a Patient with Trichorhinophalangeal Syndrome

**DOI:** 10.1155/2020/8835533

**Published:** 2020-09-11

**Authors:** Sarvie Esmaeilzadeh, Ryan S. D'Souza, Thomas M. Stewart, Matthew A. Sexton

**Affiliations:** ^1^Department of Anesthesiology and Perioperative Medicine, Mayo Clinic, Rochester, MN, USA; ^2^Department of Critical Care, Mayo Clinic, Rochester, MN, USA

## Abstract

Trichorhinophalangeal syndrome (TRPS) is a genetic disorder that may pose anesthetic challenges. We present a case of airway management for urgent surgery in a 56-year-old female with TRPS and difficult airway (macroglossia, narrow glottic opening, and hypoplastic epiglottis). Intubation was successful with video laryngoscopy using a size 2.5 pediatric blade and size 5.0 endotracheal tube. During emergence, she experienced bronchospasm and persistent urosepsis, necessitating intensive care unit (ICU) admission. Her pulmonary reserve was hindered by a Morgagni hernia causing lung compression. Our case demonstrates challenges in TRPS including challenging airway, decreased pulmonary reserve, and joint laxity introducing potential for spinal cord injury.

## 1. Introduction

Trichorhinophalangeal syndrome (TRPS) is a genetic disorder characterized by the classic triad of craniofacial, skeletal, and hair abnormalities and encompasses three subtypes (TRPS I, II, and III). Characteristic facial features include large nose with a broad ridge, underdeveloped alae, and broad eyebrows, while skeletal features include short stature, brachydactyly, and hip dysplasia [1]. TRPS type II may manifest with microcephaly, variable degrees of intellectual disability, hypermobility and laxity of joints, multiple exostoses, and redundant skin [2]. While unbiased prevalence estimates of TRPS are unavailable and likely underestimated given subtle phenotypic features, a review cites a prevalence of 0.2–1 per 100,000 people [1]. There is a paucity of published clinical data describing TRPS, particularly anesthetic management in the perioperative period. We describe a patient with known TRPS in whom urgent surgical and anesthetic intervention was required and the synergistic implications of her underlying syndrome.

## 2. Case Description

A 56-year-old female presented to the emergency department with acute-onset vomiting and severe left-sided flank pain. She reported a history of TRPS, non-ST-elevation myocardial infarction secondary to spontaneous coronary artery dissection, and reactive airway disease. While no genetic records were available characterizing her diagnosis of TRPS, she reported symptoms of “weak joints, hair loss, arthritis, and weak nails.” Surgical history included a caesarean section, hysterectomy, appendectomy, and cholecystectomy, which were uneventful. Current medications included inhaled albuterol and inhaled beclomethasone. She denied drug allergies. On examination, she was afebrile, tachycardic (129 beats/minute), tachypneic (24 breaths/minute), and had a blood pressure of 133/89 mmHg.

**Figure 1:**
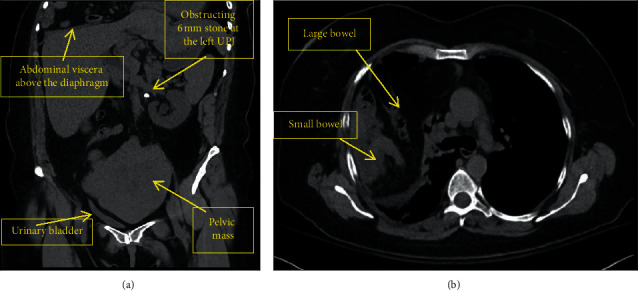
Axial (a) and coronal (b) views of CT (computed tomography) thorax, abdomen, and pelvis which revealed 6 mm left obstructing ureterolithiasis with hydronephrosis, as well as incidentally noted large 13 cm heterogeneous mass within the pelvis resulting in distortion of local structures. Additionally visualized was a large Morgagni hernia containing a large majority of the small bowel, the transverse colon, a portion of the descending colon, and a portion of the stomach with significant adjacent atelectasis of right lower lung lobe.

Complete blood count revealed mild leukocytosis (12.5 × 10^9/L). Urinalysis revealed pyuria, hemoglobinuria, proteinuria, hyaline casts, and bacteriuria, with Gram stain showing many Gram-negative bacilli. Computed tomography (CT) of the abdomen/pelvis revealed a 6 mm left ureteropelvic junction stone with hydronephrosis (Figure 1(a)). An incidental large Morgagni hernia was identified containing a majority of small bowel, the large colon, and a portion of the stomach with adjacent atelectasis of the right lower lung (Figure 1(b)). She had a large heterogenous mass in the pelvis arising from the left ovary with bladder compression and deviation to the right (Figure 1(a)). Her clinical picture was concerning for urosepsis secondary to an obstructing ureteral stone. The urological surgery team recommended urgent decompression with cystourethroscopy and left ureteral stent placement under general anesthesia. Given the evolving clinical picture of sepsis, delay of the procedure was deemed inappropriate in discussion with the surgical team.

Preanesthesia evaluation revealed an apprehensive patient with a history of severe anxiety and patient-reported history of “difficult airway” without additional available details or outside medical records elaborating on the nature of airway difficulty. The patient reported having required a “pediatric intubation set” previously. Last oral intake was three hours prior to presentation consisting of a large fatty meal. Examination revealed a female of short stature (149 cm), with a weight of 60 kg and body mass index of 27 kg/m^2^. Facial features were notable for retrognathia, microstomia, and Mallampati II. Lung auscultation revealed diffuse bilateral expiratory wheezes. Frequent rescue inhaler use was reported. Functional status was reassuring, with the patient achieving >4 metabolic equivalents daily.

The patient was unable and unwilling to tolerate awake fiber optic intubation due to her anxiety. Given the urgency of the procedure in the setting of evolving sepsis with intra-abdominal source and after full discussion of the associated risks, she was taken to the operating room with difficult airway precautions. She underwent preoperative pulmonary optimization with inhaled albuterol. Oral sodium citrate solution was ordered given aspiration risk but attempts at intake resulted in emesis. Peripheral intravenous access was established. She underwent rapid sequence induction (RSI) with manual in-line stabilization and cricoid pressure with 50 mcg fentanyl, 100 mg lidocaine, 60 mg propofol, and 70 mg succinylcholine. Initial attempt at intubation proceeded with a 3.0 video laryngoscope blade (GlideScope) and size 5.0 endotracheal tube (ETT), but ETT could not be advanced past the large blade despite a grade 1 Cormack–Lehane view. Airway was notable for macroglossia, a very narrow glottic opening, and mildly hypoplastic epiglottis. Subsequent attempt with a size 2.5 video laryngoscope blade and size 5.0 endotracheal tube was successful and atraumatic. Given full-stomach, the patient was not bag-mask ventilated between intubation attempts. Emergency rescue airway equipment was readily available including laryngeal mask airway, fiber optic scope, and cricothyrotomy kit. General anesthesia was maintained with inhaled sevoflurane and intravenous propofol infusion.

**Figure 2:**
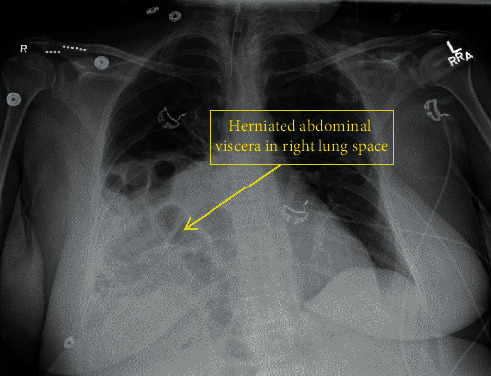
Chest X-ray revealing large right Morgagni hernia containing fat and several loops of small bowel. Associated right lung volume loss and atelectasis.

The procedure was completed uneventfully, and the anesthetic was lightened for planned extubation. However, the patient developed elevated peak pressures (>40 cm H20), became hypercapnic (end-tidal carbon dioxide (ETCO2) 56 mm Hg), and experienced a brief desaturation event to SpO2 36%. There was difficulty with ventilation and the patient was resedated, paralyzed, and given inhaled bronchodilators with improvement in ventilation and oxygenation. After improvement in bronchospasm, decision was made to transfer the patient to ICU due to reduced pulmonary reserve and ongoing urosepsis. At this time, peak pressures remained >30 cmH20, while on FiO2 of 50% on continuous mandatory ventilation-volume control and tidal volume of 300 cc. Chest X-ray revealed an appropriately positioned endotracheal tube (Figure 2). Decision was made to keep the patient intubated overnight while her sepsis resolved, and she was uneventfully extubated on postoperative day 1. To reduce aspiration risk, a nasogastric tube was placed to suction gastric contents prior to emergence both postsurgically and in the ICU.

## 3. Discussion

We present the challenging case of a patient with TRPS and difficult airway who presented for urgent surgery. She was successfully intubated but experienced intraoperative bronchospasm, requiring prolonged intubation and unanticipated admission to the intensive care unit (ICU).

Our preanesthetic evaluation revealed retrognathia, microstomia, and a history of difficult airway. These physical exam characteristics are consistent with prior reports of patients with TRPS [3]. Unfortunately, our patient was unable to provide details regarding the facility where her previous difficult airway was encountered and was additionally unable to clarify the state in which she had received care, thus limiting our ability to evaluate prior records. Thorough discussions were undertaken with the patient regarding the relative safety of awake fiber optic intubation in the setting of her difficult airway history; however, significant sedation in the setting of her recent oral intake was felt to be contraindicated, and the patient refused consent for true awake fiber optic. Given the patient's severe anxiety, NPO (nil per os) status, and refusal for awake intubation, but persistent aspiration risk, we elected for RSI and intubation via video laryngoscopy, but with preparation of back-up rescue airway equipment, as well as supraglottic airway devices, specifically size 3 and 4 laryngeal mask airways. Prior to proceeding to the operating room, discussion was undertaken with the otolaryngologist team on call regarding the potential need for emergent surgical airway. The team evaluated the patient and felt that despite a short neck, her anatomy was easily amenable to emergent airway if needed. They remained on emergent back-up call.

The use of nondepolarizing muscle relaxant was not considered in this case, as a reversal agent, i.e., sugammadex (which could potentially be required in the setting of a cannot-intubate-cannot-ventilate situation), was not available at our institution at the time. Fentanyl, though not typically employed in RSI, was used at induction to minimize hemodynamic shifts in anticipation of possible repeated attempts at securing the airway.

Preinduction, given aspiration risk, consideration was made for use of the prokinetic agent metoclopramide, but in the setting of a known large Morgagni hernia, we felt the benefits did not outweigh the risk of potential visceral strangulation or incarceration. We elected to pursue the use of nonparticulate oral sodium citrate, but unfortunately the patient was unable to tolerate this and developed further nausea and vomiting. Manual in-line stabilization was performed because of potential presence of cervical spine hypermobility and laxity in TRPS introducing the theoretical risk for cervical spinal cord injury [3]. A smaller-sized laryngoscope blade likely allowed better visualization of the epiglottis due to the presence of limited submandibular space that often accompanies micrognathia and retrognathia, but despite adequate visualization of the glottis with a 3.0 sized blade, the 5.0 ET tube could not be advanced. Decision was made to downsize to 2.5 sized blade in order to allow adequate space, such that the ET tube could pass adjacent to the blade. We speculate that since TRPS patients may have altered pharyngeal and laryngeal structures including macroglossia, narrow glottic opening, and epiglottic aplasia, they may warrant smaller-sized endotracheal tubes and laryngoscope blades [4]. In situations where patients are willing and NPO guidelines are met, these patients may warrant consideration for awake or sedated fiber optic intubation.

During emergence, the patient experienced desaturation with elevated ETCO2 and peak pressures, presumably due to bronchospasm. Predisposing factors included a history of asthma, active smoking, preoperative examination revealing expiratory wheezes, airflow resistance through a smaller endotracheal tube, and suboptimal oxygen utilization from urosepsis. While our patient was afebrile and denied a cough, studies have reported frequent respiratory infections in TRPS, a predilection which is only further exacerbated given her history of chronic tobacco use [5]. Importantly, the presence of a large Morgagni hernia compressing the right lung and leading to right lower lung atelectasis likely decreased this patient's functional residual capacity (FRC), thus making her susceptible to hypoxemia. Morgagni hernias are rare, comprising only 2% of all diaphragmatic hernias, and have not been previously reported in TRPS. While uncommon, Morgagni hernias have the potential to cause significant morbidity if diagnosis is missed [6].

We speculate the subtype of TRPS that characterized our patient's phenotype. While this is a diagnosis confirmed with genetic mutation analysis, our patient did not have genetic records available. The authors believe that type II TRPS, also known as Langer–Giedion syndrome, likely characterized this patient's phenotype. Type II TRPS is secondary to 8q24.11 microdeletion [2]. Its phenotype includes short stature, elongated philtrum, bulbous nose, thin upper lip, thin and sparse hair, receding mandible, abnormal dentition, cone-shaped epiphyses, redundant skin, hypotonia, bony exostoses, recurrent respiratory and urinary tract infections, and intellectual disabilities [7].

A review of the literature revealed two published reports of airway management in patients with TRPS—an 8-year-old male with TRPS II in whom a supraglottic airway was successful [3] and a 14-year-old female with TRPS I and III who was intubated by direct laryngoscopy [5]. These published cases also noted distinctive facial features, distorted upper airway anatomy, and abnormal dentition, which may impact laryngoscopy and mask ventilation. Furthermore, TRPS type II patients may express hypertrophied nasal alae (with implications for nasal airway), epiglottic aplasia, cleft palate, difficulty in peripheral intravenous cannulation due to redundant skin, and poor patient cooperativity in the setting of intellectual disabilities.

In summary, we present a case of TRPS with successful RSI and intubation of an adult female using video laryngoscopy. She experienced bronchospasm postoperatively necessitating ICU admission. Her baseline anatomy related to TRPS requiring smaller-sized endotracheal tube in conjunction with asthma and pulmonary atelectasis in the setting of a large diaphragmatic hernia resulted in desaturation and difficulty with ventilation. This case demonstrates the role of a decreased baseline pulmonary reserve in patients diagnosed with TRPS, as well as other potential anesthetic challenges related to hypermobility and laxity introducing potential for spinal cord injury, anemia increasing risk for reduced oxygen delivery, and excessive skin impeding intravenous access.

## Data Availability

No data were used to support this study.
